# Supplementary material for: Mesenchymal Stem/Stromal Cells Derived from Dental Tissues Mediate the Immunoregulation of T Cells through the Purinergic Pathway
Source: Int J Mol Sci. 2024 Sep 4;25(17):9578. doi: 10.3390/ijms25179578 (PMC11395442; doi:10.3390/ijms25179578)
Supplement: Supplementary file 1 [file ijms-25-09578-s001.zip › Supplementary Figure S1.pdf]

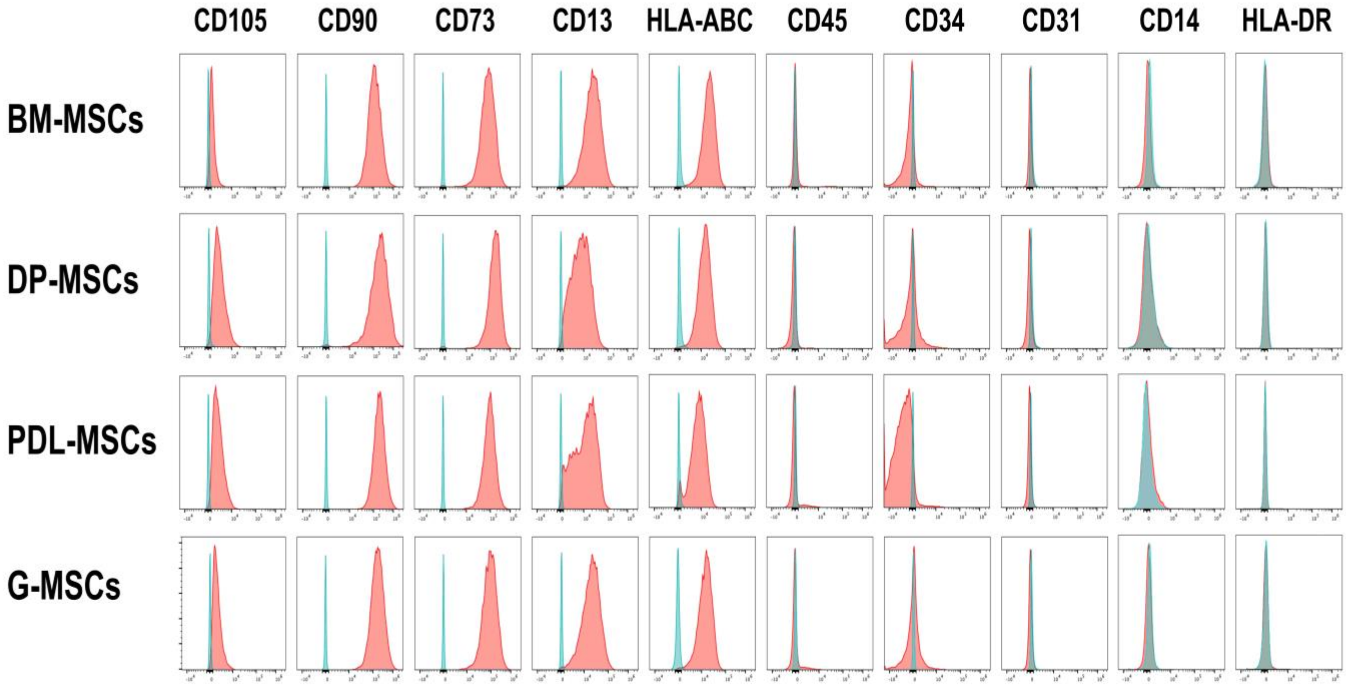

Supplementary Figure S1. Immunophenotypic profile of dental tissue samples. Representative histograms of the expression of specific markers in MSCs obtained from all samples (n = 9 replicates/source).
