# Supplementary material for: Mesenchymal Stem/Stromal Cells Derived from Dental Tissues Mediate the Immunoregulation of T Cells through the Purinergic Pathway
Source: Int J Mol Sci. 2024 Sep 4;25(17):9578. doi: 10.3390/ijms25179578 (PMC11395442; doi:10.3390/ijms25179578)
Supplement: Supplementary file 1 [file ijms-25-09578-s001.zip › Supplementary Figure S2.pdf]

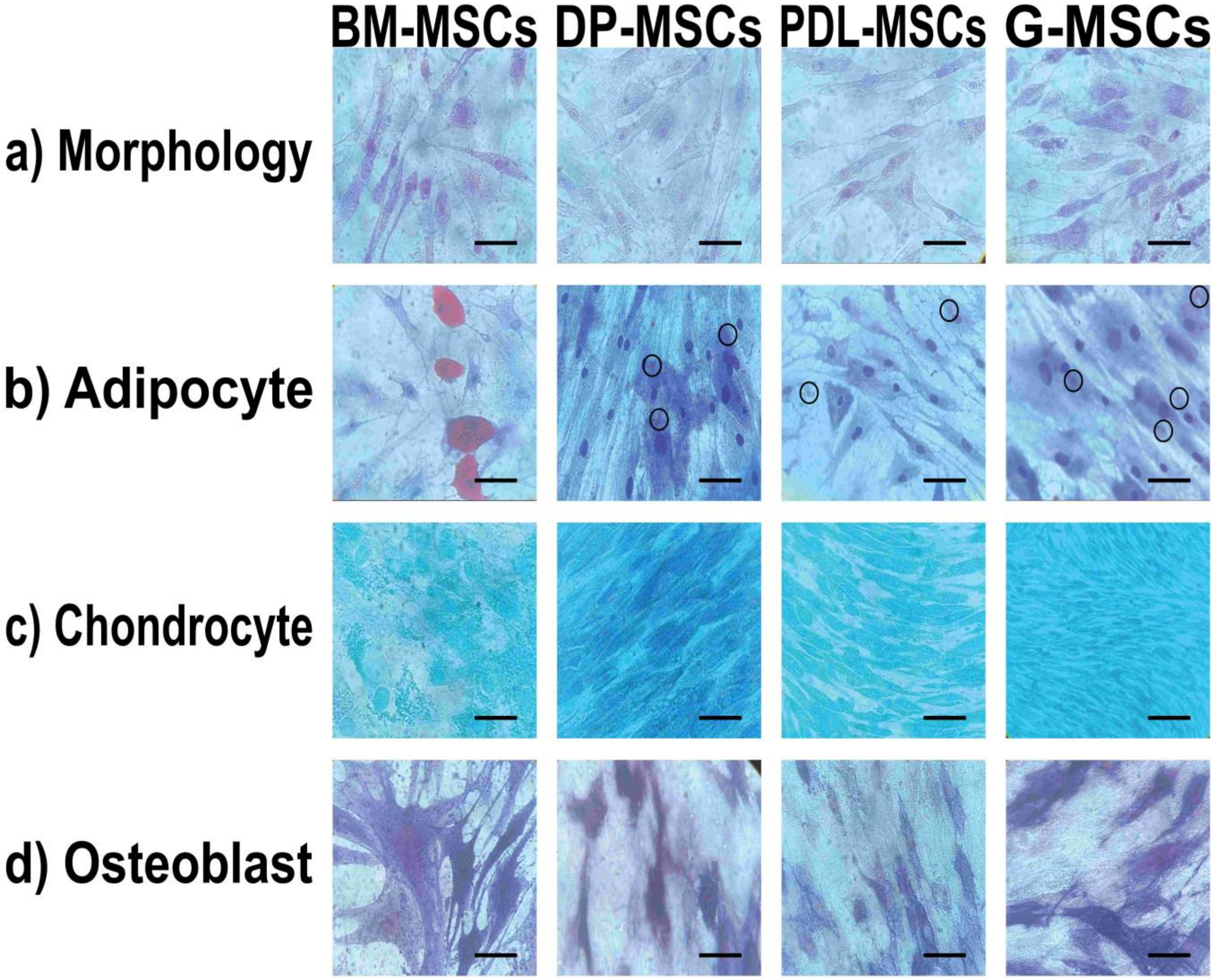


Supplementary Figure S2. Morphology and differentiation capacity of dental tissue samples. Rep- resentative images of a) fibroblast morphology of dental tissue samples under standard culture conditions and stained with toluidine blue (bar size 100 µm) and their ability to differentiate in a specific inducing medium into b) adipocytes (bar size 200 µm), c) chondrocytes (bar size 100 µm) and d) osteoblasts (bar size 100 µm), as revealed by oily red, alcian blue and alkaline phosphatase staining, respectively. (n = 9 repetitions/source).
