# Supplementary material for: Mesenchymal Stem/Stromal Cells Derived from Dental Tissues Mediate the Immunoregulation of T Cells through the Purinergic Pathway
Source: Int J Mol Sci. 2024 Sep 4;25(17):9578. doi: 10.3390/ijms25179578 (PMC11395442; doi:10.3390/ijms25179578)
Supplement: Supplementary file 1 [file ijms-25-09578-s001.zip › Supplementary Table S1.pdf]

Supplementary Table S1. Percentage of DT-MSCs expressing immunophenotypic markers

Marker/Source	BM-MSCs	DP-MSCs	PDL-MSCs	G-MSCs
CD105	88.87 ± 0.80	95.57 ± 1.08	97.07 ± 1.71	91.83 ± 4.50
CD90	97.57 ± 1.50	97.63 ± 1.16	99.50 ± 0.36	99.27 ± 0.12
CD73	99.23 ± 0.35	98.87 ± 0.29	99.20 ± 0.52	99.87 ± 0.06
CD13	99.63 ± 0.12	95.63 ± 5.20	96.40 ± 0.95	99.67 ± 0.31
HLA-ABC	89.40 ± 13.65	91.03 ± 6.90	94.17 ± 0.12	97.77 ± 1.31
CD45	0.42 ± 0.12	0.63 ± 0.96	0.37 ± 0.44	0.53 ± 0.32
CD34	2.00 ± 1.58	0.69 ± 0.56	0.47 ± 0.20	1.29 ± 0.18
CD31	1.13 ± 1.21	0.91 ± 0.80	0.65 ± 0.76	0.75 ± 0.85
CD14	0.31 ± 0.02	0.93 ± 0.23	0.92 ± 0.84	0.80 ± 0.85
HLA-DR	0.62 ± 0.56	0.31 ± 0.22	0.67 ± 0.55	0.74 ± 0.63

The values are shown as percentages of positive cells for the different markers and correspond to the average of the values from independent experiments (n= 9 repetitions/source).
